# Is cardia cancer a special type of gastric cancer? A differential analysis of early cardia cancer and non-cardia cancer

**DOI:** 10.7150/jca.51433

**Published:** 2021-03-01

**Authors:** Liting Lv, Xiao Liang, Dan Wu, Feng Wang, Yan Zhang, Hui Cang, Xiongwei Deng, Mei Li

**Affiliations:** 1 Department of oncology, Affiliated hospital of Nantong University, Nantong, 226001, Jiangsu Province, China.; 2 Department of oncology, Jiangyin People's Hospital, Wuxi, 214400, Jiangsu Province, China.; 3 Department of gastroenterology, Jiangyin People's Hospital, Wuxi, 214400, Jiangsu Province, China.; 4 Department of orthopedics, Jiangyin People's Hospital, Wuxi, 214400, Jiangsu Province, China.

**Keywords:** early gastric cancer, cardia cancer, non-cardia caner, prognosis, propensity score matching.

## Abstract

**Background:** The prognosis of early cardia cancer and non-cardia cancer is still controversial. It is difficult to collect a large number of cases with complete information in clinical practice. Our study was aimed to identify the differences in clinicopathological characteristics and outcomes of early cardia gastric cancer and non-cardia gastric cancer.

**Methods:** All cases analyzed were from Surveillance, Epidemiology, and End Results database. The data of the patients with early gastric cancer from 2004 to 2010 was retrospectively analyzed. Patients were distributed to cardia cancer group and non-cardia cancer group. Univariate and multivariate analyses were performed to examine differences between groups. The competitive risk model was made to compare the association with cardia cancer and non-cardia cancer about the causes of death. Propensity score matching (PSM) was performed to reduce the bias.

**Results:** We found that cardia cancer was more common in male patients and the White than that in non-cardia cancer at early stage, signet ring cell carcinoma was more common in non-cardia cancer, and the differentiation of non-cardia cancer was worse. Univariate analysis showed that age, marital status, race, tumor location, histology, grade, stage, and operation or not can determine the prognosis. And the prognosis of patients with cardia cancer was worse than that of non-cardia cancer, according to lymph node metastasis and the depth of tumor invasion. Multivariate analysis showed cardia cancer was an independent prognostic factor for poor prognosis. After PSM, cardia cancer still exhibited poor prognosis.

**Conclusions:** At early stage, cardia cancer had a poor prognosis compared with non-cardia cancer. The prevention and treatment of early cardia cancer need to be seriously treated.

## Introduction

Gastric cancer is one of the most common cancers and the third leading cause of cancer-related death in the world 1. About 1 million cases of gastric cancer are newly diagnosed every year recently 2, 3. In the past few decades, the mortality of gastric cancer has decreased significantly in many countries. However, gastric cancer is still a disease with poor prognosis and high mortality. Most patients are in the middle or late stage when diagnosed, with very short survival and high mortality, which can't be cured 4. The 5-year survival rate of early gastric cancer in Japan is more than 90% 5, while that of advanced gastric cancer is 14%~25% 6. Early diagnosis and surgical resection of gastric cancer provide the best treatment for patients with gastric cancer, and prolong their survival, thus early diagnosis is particularly important 7. The World Health Organization (WHO) defined early gastric cancer (EGC) as gastric cancer confined to the gastric mucosa or submucosa, regardless of the lymph node metastasis 8, 9. A low EGC diagnosis rate and high postoperative recurrence rate are still major obstacles in gastric cancer therapy. Because the number of cases is limited, studies targeted at EGC are relatively limited.

The two main sites of gastric cancer are proximal gastric (cardia) and distal gastric (non-cardia)^10^. Although the incidence rate of non-cardia cancer has declined in Western countries, that of cardia cancer has been increasing since 1970s 11, 12. Epidemiological studies show that cardia cancer has different features, like geographical location, race, socioeconomic status, incidence rate and prognosis, compared with non-cardia cancer 13. Non-cardia cancer is common in developing countries, blacks and people with low socioeconomic status, while cardia cancer is more common in developed countries, white people and people with higher socioeconomic status. Helicobacter pylori (HP) infection and dietary factors play an important role in the occurrence and development of non-cardia cancer 14, While the main risk factors of cardia cancer include Gastroesophageal reflux disease and obesity 15. The incidence rate of gastric cancer varies with its location, which indicates that cardia cancer and non-cardia cancer may represent two different etiological diseases.

The survival rate of gastric cancer in countries with high incidence rate is better than that countries with low incidence rate, largely due to the difference in survival rate based on the location of gastric tumors 16. Cardia cancer and non-cardia cancer have different biological characteristics in populations of different geography, ethnicity and socio-economy 13. A recent Chinese data shows that cardia cancer is more likely to lymphoid stroma than non-cardia cancer, owing to greater tendency toward submucosal invasion 17. It is difficult to carry out a large number of samples based on early gastric cancer. In recent years, epidemiology has found that the incidence rate of cardia cancer is increasing, which has attracted many specialists' attention. Many researchers believe that cardia cancer and non-cardia cancer may belong to two different types of tumors with different clinical and pathological characteristics 18. However, the prognosis of early cardia cancer and non-cardia cancer is controversial. Some studies have reported a worse prognosis in patients with cardia cancer, while others have found no significant differences 19, 20. Katsuhiko et al. even found that patients with cardia cancer had a long-term survival rate than patients with non-cardia cancer 21.

Surveillance, Epidemiology, and End Results (SEER) is a large caner database in the United States, covering one third of the cases in the United States. SEER currently captures 400000 cancer cases a year and collects cancer diagnosis, treatment, and survival data for about 30% the U.S. population 22. Therefore, this study is based on SEER database to analyze the clinicopathological characteristics and prognosis of patients with early cardia cancer and non-cardia cancer, and to help patients choose the best treatment.

## Patients and Methods

### Patients selected

**Figure 1:**
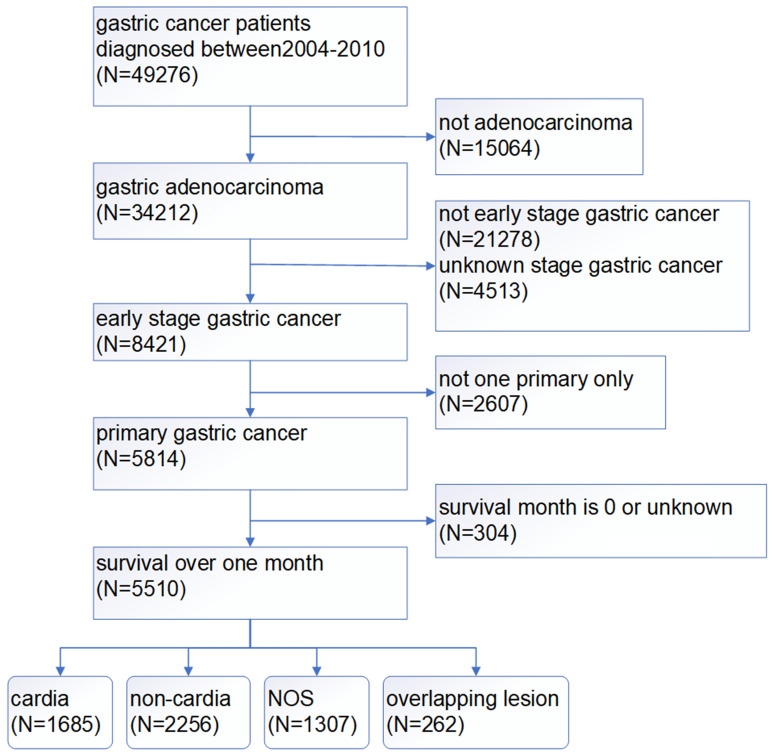
The screening process of patients with gastric cancer.

**Figure 2:**
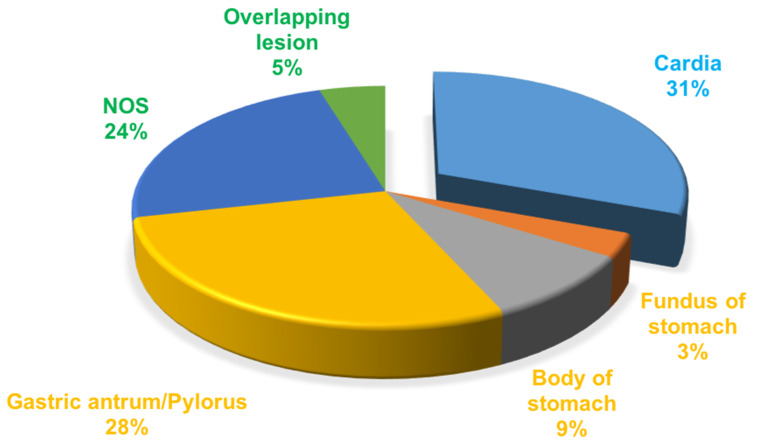
The proportion of the sites in gastric cancer.

A total of 49276 patients were diagnosed with gastric cancer from 2004 to 2010 in the database. 34212 patients of those were confirmed by the ICD as adenocarcinoma (8140/3: Adenocarcinoma, Not Otherwise Specified (NOS); 8144/3: Adenocarcinoma, intestinal type; 8145/3: Carcinoma, diffuse type; 8211/3: Tubular adenocarcinoma; 8260/3: Papillary adenocarcinoma, NOS; 8480/3: Mucinous adenocarcinoma; 8490/3: Signet ring cell carcinoma). The treatment of gastric cancer is mainly guided by the TNM staging, so we choose stage I as early gastric cancer to carry on research based on AJCC 6th TNM staging. According to the screening process as follows (Figure 1), patients with early gastric cancer were divided into four groups: cardia cancer, non-cardia cancer, not otherwise specified gastric cancer and gastric cancer with overlapping lesions. Non-cardia cancer included fundus of stomach (C16.1-Fundus of stomach), body of stomach (C16.2-Body of stomach), gastric antrum (C16.3-Gastric antrum) and pylorus (C16.4-Pylorus). NOS included lesser curvature of stomach without particular sites (C16.5-Lesser curvature of stomach NOS), greater curvature of stomach without particular sites (C16.6-Greater curvature of stomach NOS) and gastric cancer without particular sites (C16.9-Stomach, NOS). Specific percentages of sites were shown in Figure 2. NOS group and overlapping lesion were excluded without specific sites. 1685 patients with early cardia cancer and 2256 patients with non-cardia cancer were included in the study. Inclusion criteria: patients diagnosed with gastric cancer between 2004~2010; with a definite tumor site; gastric adenocarcinoma; early stage gastric cancer; primary gastric cancer; patients with the survival over one month. Exclusion criteria: not adenocarcinoma, not early stage gastric cancer, unknown stage gastric cancer, not one primary only, patients whose survival month is 0 or unknown, NOS, overlapping lesion. The year 2004 was selected as the first year of this study because relatively detailed information was provided after 2004. The year 2010 was selected as the last year of this study to ensure at least 5-year follow-up time.

The main endpoint of the study was cancer specific survival (CSS) which was the time from the date of diagnosis to the date of death caused by gastric cancer. The secondary endpoint was overall-survival (OS) which was the time from the date of diagnosis to the date of death.

### Statistical analysis

All data analyses were performed using SPSS, version 23.0 (SPSS, IL, USA) and R 3.6.3. All pictures were drawn by GraphPad Prism 7.0. Chi-square tests were used to compare the differences between varieties. Kaplan-Meier models and log-rank tests were used for the univariate analysis of survival. Cox regression was used for multivariate analysis. R package MatchIt was used for propensity score matching (PSM). R package cmprsk was used for competitive risk model. *P* value less than 0.05 was set for the significant meaning.

## Result

### Epidemiological trends

**Figure 3:**
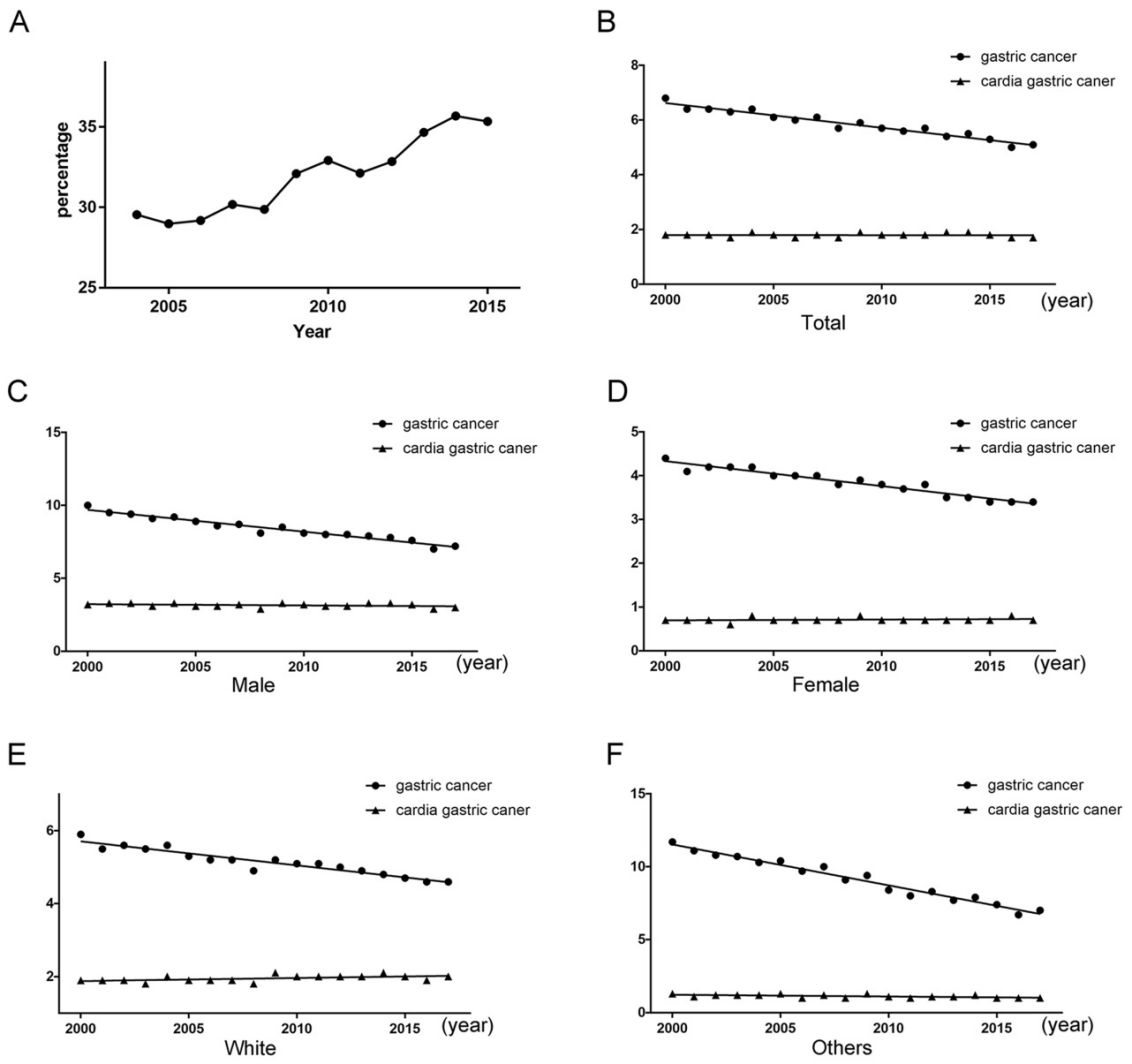
(A) Tendency about the proportion of cardia gastric cancer patients from all gastric cancer patients. (B) Tendency about rates of per 100000 and age-adjusted to population. (C) Tendency about rates of per 100000 and age-adjusted to population for male. (D) Tendency about rates of per 100000 and age-adjusted to population for female. (E) Tendency about rates of per 100000 and age-adjusted to population for female for the white race. (F) Tendency about rates of per 100000 and age-adjusted to population for the other races.

The patients with gastric cancer from 2004 to 2015 were collected for this study. The share of cardia gastric cancer had an increasing tendency about five percentage points from 2004 to 2015 (Figure 3). The rates of per 100000 and age-adjusted to population for gastric cancer exhibited a declining tendency (*P* < 0.001), but the rates for cardia gastric cancer still exhibited a stable tendency (*P* = 0.929). The prevention and treatment of cardia cancer need to be seriously treated.

### Baseline characteristics of patients

A total of 3941 patients were included in the study. The baseline characteristics were shown in Table 1. Among the 3941 patients, 1685 patients (42.8%) were diagnosed as cardia cancer. 2256 patients (57.2%) were diagnosed as non-cardia cancer. Non-cardia cancer seemed to have less differentiation degree and worse pathologic type than cardia cancer. Non-cardia cancer had higher proportion of poor differentiation or undifferentiated cancer (Chi-square = 51.272, *P* < 0.001, Table 1) and signet ring cell carcinoma than cardia cancer (Chi-square = 80.28, *P* < 0.001, Table 1).

### Prognostic factors of patients with early gastric cancer

**Figure 4:**
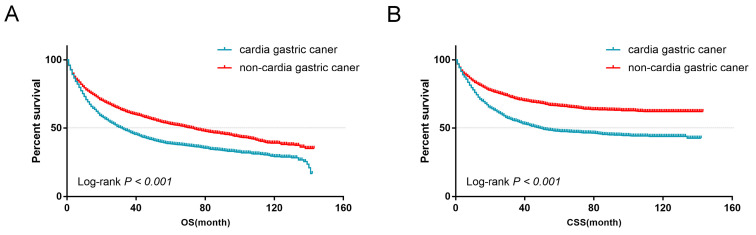
(A) OS for patients with cardia cancer and non-cardia cancer. (B) CSS for patients with cardia cancer and non-cardia cancer.

**Figure 5:**
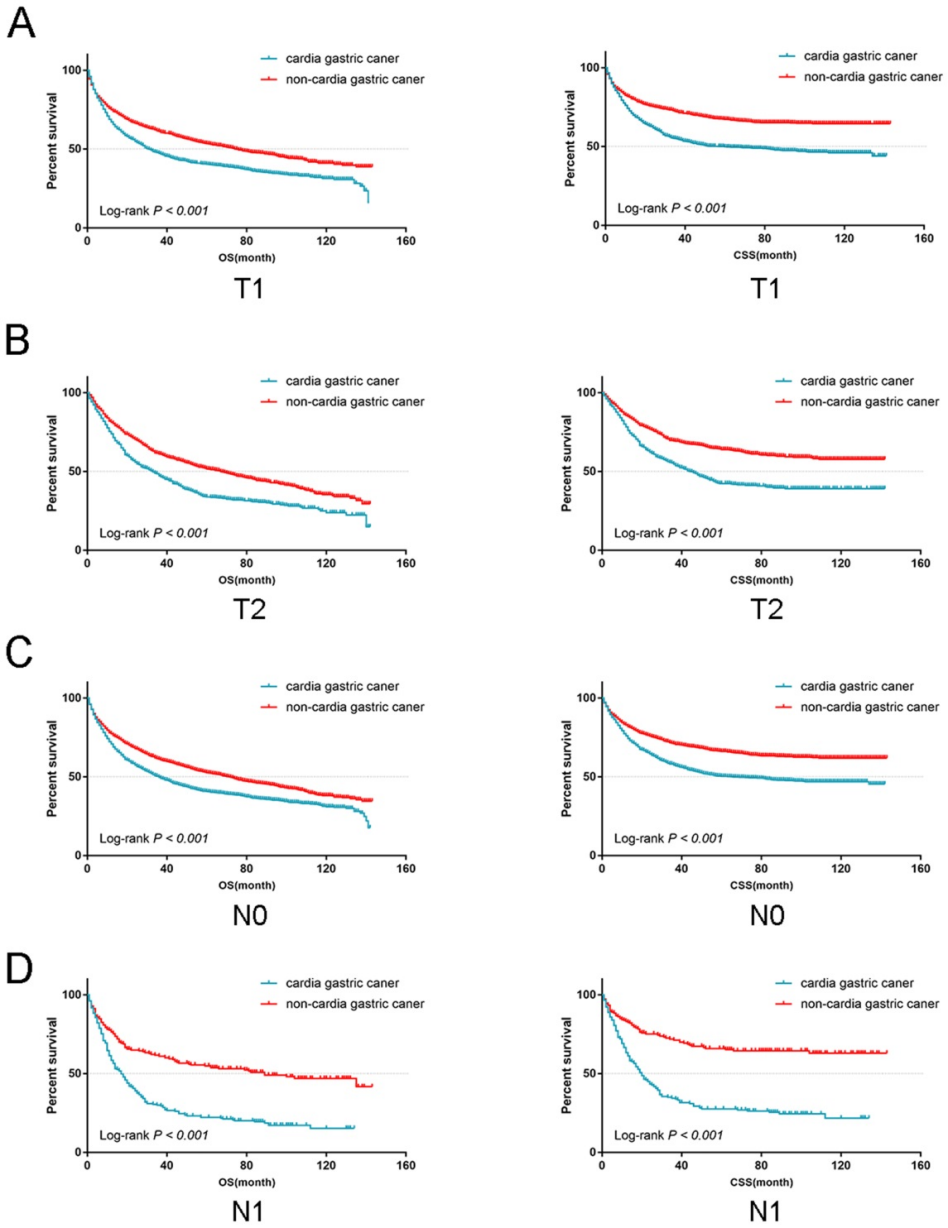
The OS and CSS for the patients with cardia cancer and non-cardia cancer according to lymph node metastasis and the depth of tumor invasion, respectively.

From the univariate analysis, we found the site of gastric cancer was the predictor of OS (Log-rank *P* < 0.001, Figure 4A) and CSS (Log-rank *P* < 0.001, Figure 4B). Cardia cancer had a poor prognosis. The one-, two-, and five-year OS rates for cardia cancer were 68.8%, 54.9%, and 38.2%, respectively, while the one-, two-, and five-year OS rates for non-cardia cancer were 78.2%, 69.1%, and 54.3%, respectively. The one-, two-, and five-year CSS rates for cardia cancer were 74.4%, 61.9%, and 47.6%, respectively, while the one-, two-, and five-year CSS rates for non-cardia cancer were 83.5.2%, 76.9%, and 67.5%, respectively. By multivariate analysis of CSS, cardia cancer was an independent predictor of poor prognosis (cardia cancer vs non-cardia cancer, Hazard ratio (HR) = 0.806, 95% CI = 0.719~0.904, *P* < 0.001, Table 2). Considered that different TN stages had various prognosis, we further analyzed the prognosis according to different T and N states. Univariate analysis showed that the prognosis of early cardia cancer was worse than that of early non-cardia cancer, regardless of T1, T2 or lymph node metastasis (Log-rank* P* < 0.001, Figure 5).

### The cumulative incidence function curve

**Figure 6:**
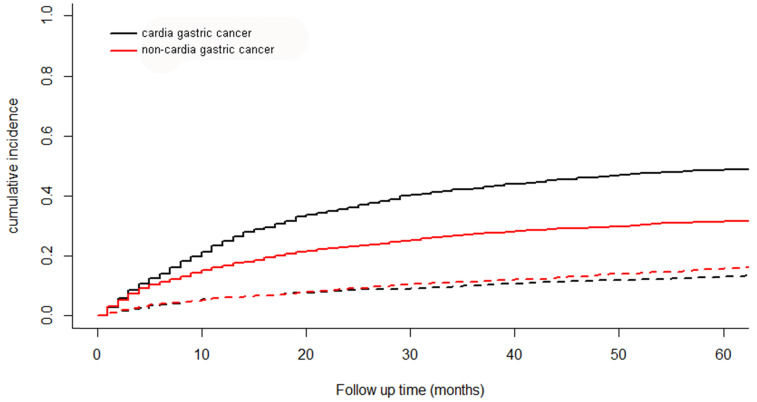
The cumulative incidence function curve for cardia cancer and non-cardia cancer. Solid line represents cause-specific death and dotted line represents other cause of death.

A total of 2372 patients died during the follow-up period. 1597 patients were from cancer specific death and 775 from other causes. The cumulative incidence function curve for cardia cancer and non-cardia cancer was shown in Figure 6. The 5-year cumulative incidence of cancer-specific mortality for cardia cancer was 48.6% and that for non-cardia cancer was 31.5%.

### Subgroup analysis

**Figure 7:**
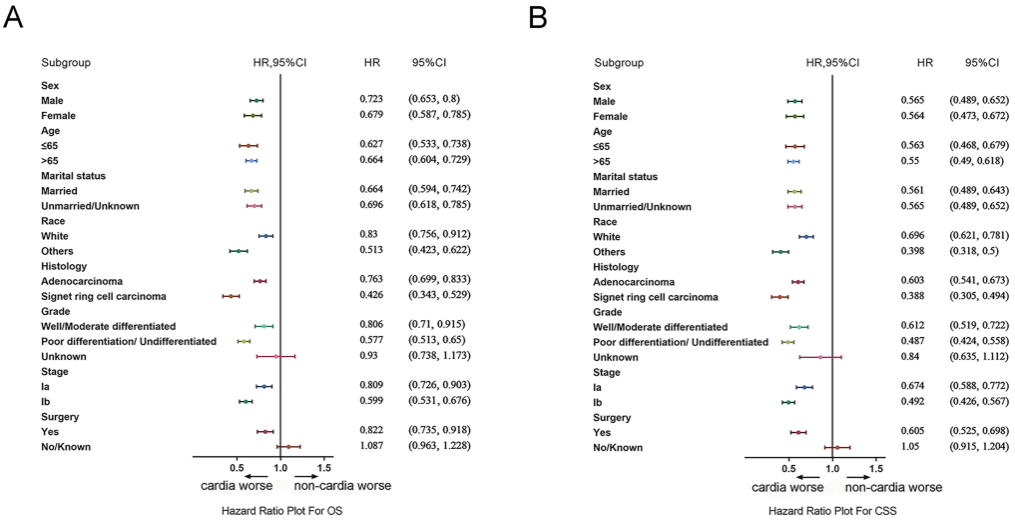
(A) Forest plots of HRs by the COX model for OS between patients with early cardia cancer and non-cardia cancer. (B) Forest plots of HRs by the COX model for CSS patients with between cardia cancer and non-cardia cancer.

The differences of the prognosis between cardia cancer and non-cardia cancer were analyzed in the subgroup analysis. Forest plots of HRs by the COX model were shown in Figure 7A and 7B. In the forest plots of both OS and CSS, the prognosis for patients with all genders, all age groups, all races, all stages, and pathologic types were worse in cardia cancer. In the subgroup of operation group, patients with cardia cancer were also shown poor prognosis than the patients with non-cardia cancer. Only in the subgroup of conservative group (cardia cancer vs non-cardia cancer: OS, HR = 1.087, 95% CI = 0.963~1.228; CSS, HR = 1.05, 95% CI = 0.915~1.204) and patients with unknown grade (cardia cancer vs non-cardia cancer: OS, HR = 0.93, 95% CI = 0.738~1.173; CSS, HR = 0.84, 95% CI = 0.635~1.112) were not significantly different between patients with cardia cancer and non-cardia cancer.

### PSM and survival analysis in matched groups

**Figure 8:**
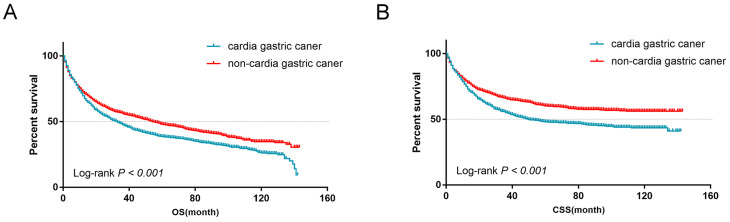
(A) OS for patients with early cardia cancer and non-cardia cancer after PSM (B) CSS for patients with early cardia cancer and non-cardia cancer after PSM.

A PSM analysis was performed to adjust the differences in clinical characteristics at a ratio of 1:1 and a caliper of 0.01. After PSM, 1069 patients with non-cardia cancer were matched with 1069 patients with cardia cancer from 1685 patients with cardia cancer. Similar basic clinical characteristics were observed between the cardia cancer group and the non-cardia cancer group after matching (Table 3). There were no significant differences in the gender group (*P* = 0.742), age group (*P* = 0.244), marital status group (*P* = 0.289), race group (*P* = 0.954), histology group (*P* = 0.429), grade group (*P* = 0.304), stage group (*P* = 0.272) and surgery group (*P* = 0.640). After PSM, patients with non-cardia cancer showed a more favorable outcome, while patients with cardia cancer showed a worse prognosis in terms of OS (Log-rank *P* < 0.001, Figure 8A) and CSS (Log-rank *P* < 0.001, Figure 8B).

## Discussion

Gastric cancer is a cancer with high incidence and early physical examination in high-risk areas can reduce mortality 15. The 5-year survival rate of early gastric cancer is about 95% 23. Large-scale screening has led to a decline of mortality about gastric cancer in Japan, while the five-year relative survival rate is less than 20% because of lack of early detection in the United States 15. About 90% gastric cancer is adenocarcinoma, and its histology is divided into two types: highly differentiated or intestinal type, and undifferentiated or diffuse type 15. In recent years, the main reason for the decline of the incidence of gastric cancer worldwide is mainly due to the decline in the incidence of intestinal tumors in gastric body 24. However, the incidence of diffuse gastric cancer is increasing, especially signet-ring gastric cancer 25. According to the location, gastric cancer can be divided into cardia and non-cardia cancer. Some scholars believe that the incidence of cardia cancer is gradually increasing while that of non-cardia cancer is decreasing 15.

Cardia cancer occurs in the narrow area of the proximal stomach below the gastroesophageal junction (GEJ) 12, 26. Non-cardia cancer is associated with Helicobacter pylori infection, while the pathogenesis of cardia cancer remains unclear 8. Many investigators have demonstrated the similarities between cardia cancer and distal esophageal adenocarcinoma (EAC) in terms of epidemiology, molecular pathology, clinical features and survival. Western researchers believe that cardia cancer is a part of the EAC spectrum, because cardia cancer occurs in the proximal gastric myocardial mucosa within 3 cm below the gastroesophageal junction, and may originate from the short segment Barrett esophagus 27, 28. Ethnic and geographic differences related to the incidence of cardia cancer worldwide imply that the pathogenesis of cardia cancer and non-cardia cancer is different. And it is speculated that the two tumors may have different sources and are distinct diseases.

Our studies have found that cardia cancer occurs mainly in the male and white patients (Table 1). A study in Japan found that smoking is an important risk factor for cardia cancer 29. Most studies have not found alcohol consumption to be an important risk factor for cardia cancer 15, 30. In addition, Helicobacter pylori is a risk factor for non-cardia cancer, but not an independent risk factor for cardia cancer 8. Other environmental toxins, such as industrial pollution, have also been identified as an independent risk factor 31. Lack of fresh vegetables or fruits, and foods and diets in high-salt and obesity were not considered independent risk factors for cardia cancer 18. Our study also found that patients of early cardia cancer had a worse prognosis than non-cardia cancer (Figure 4A and 4B), which was similar to a Chinese study. We found that signet-ring cell carcinoma of early gastric cancer had a better prognosis than adenocarcinoma, which is consistent with previous findings 32. In recent years, many studies have revealed the molecular characteristics of cardia cancer. Compared with non-cardia cancer, the expression of *HER2, Sirt1* and* TP53* genes in cardia cancer was significantly increased, and *PAKI* and *KRAS* genes were significantly amplified, indicating a poor prognosis 33-35. Therefore, some scholars believe that cardia cancer and non-cardia cancer are two different entities.

In our study, 3941 patients with early gastric cancer classified as cardia cancer and non-cardia cancer were collected from SEER database. Our study showed that the incidence rate of cardia cancer was increasing. Although the detection ratio of early stage was high, the prognosis of cardia cancer was still poor even with better types and differentiation. According to the 6^th^ TNM staging, we classified the stage I gastric cancer as early gastric cancer, including T1N0, T1N1 and T2N0 stages. We used univariate analysis to discuss the prognosis in regarding to T1, T2, N0 and N1 stages, separately. Through data analysis, we found that patients with early cardia cancer had worse prognosis than patients with early non-cardia cancer (Figure 5). Besides, In the forest plots, the subgroup of conservative group and patients with unknown grade were not significantly different between early cardia cancer group and non-cardia cancer group. However, the unknown grade group was not common and the group was included few patients, thus the result might exist bias. Besides, most patients would be operated when diagnosed with early gastric cancer. The patients without operation due to other reasons were out of our discussion. The treatment methods are mainly by endoscope or operation for early gastric cancer. In China, endoscopic resection, like Endoscopic submucosal dissection (ESD), has gradually become a new option for early gastric cancer 36. The 5-year survival rate of patients with early gastric cancer after surgery (or endoscopic resection) can reach 90%~95% 8. Many studies were to explore the different clinicopathological characteristics between cardia cancer and non-cardia cancer about advanced gastric cancer in clinical stage from 2~4 8, 37. The definition of early gastric cancer is controversial. In the fourth edition, WHO defined early gastric cancer as a cancer with definite invasion, which was limited to the mucosa and submucosa, regardless of lymph node metastasis 38. However, the definition of early gastric cancer has been deleted in the fifth edition. In our study, we choose stage I as early gastric cancer, because the clinical therapy depends on the TNM staging. The 5-year survival rate of our study was lower than other studies, because it was a population-based study. The one- and five-year CSS rates for patients undergoing surgeries in our study were 90.9% and 73.3%. In addition, cardia cancer cases were paired with non-cardia cancer cases according to factors like age, race and sex to minimize the impact of selectivity bias on our study results, which showed that cardia cancer patients had a worse prognosis than patients with non-cardia cancer after PSM (Figure 8A and 8B). Most cardia cancer have infiltrated deep into the stomach and metastasized to lymph nodes and distant organs 39, 40. Therefore, in the early stage, we recommend careful preoperative evaluation and consideration of the best surgery for cardia cancer to reduce the mortality of cardia cancer.

Signet ring cell carcinoma of early non-cardia cancer was more common than that in cardia cancer. This was consistent with some research data 41. However, some conclusions were controversial, possibly because different regions and countries might have different types of tumor differentiation. This study was a retrospective study with limitations. Some important messages such as surgical reasons and chemotherapy were not analyzed. Besides, the PS score was not involved in the database. We didn't know the general condition and recovery of the patients. What's more, missing data and potential coding errors might affect our analysis. Early stage cancer nearly seldom affects the performance status of patients. The PS score is not an important indicator in the study of early stage gastric cancer. In the inclusion criteria, the patients with follow-up time of 0 were deleted. The deletion of patients with short-term death after surgery could largely exclude the bias to some extent, thus the data would be more reliable. In summary, our data suggested that cardia cancer might exhibit different clinicopathological characteristics than non-cardia cancer. The genetic and molecular characteristics of cardia cancer still need future research. With the implementation of TCGA and whole genome sequencing, we might explore the molecular typing in the future.

## Conclusions

At early stage, cardia cancer had a poor prognosis compared to non-cardia cancer, although non-cardia cancer seemed to have less differentiation degree and worse pathologic type than cardia cancer. The prevention and treatment of cardia cancer need to be seriously treated.

## Figures and Tables

**Table 1:** Clinical characters for patients with early cardia cancer and non-cardia cancer

	N	cardia	non-cardia	chi-square	*P*
**Gender**				231.401	<0.001*
Male	2504	1298	1206		
Female	1437	387	1050		
**Age**				33.258	<0.001*
<65	1388	679	709		
≥65	2553	1006	1547		
**Marital status**				41.382	<0.001*
Married	2286	1076	1210		
Unmarried/Unknown	1655	609	1046		
**Race**				525.673	<0.001*
White	2798	1500	1298		
Others	1143	185	958		
**Histology**				80.28	<0.001*
Adenocarcinoma	3337	1527	1810		
Signet ring cell carcinoma	604	158	446		
**Grade**				51.272	<0.001*
Well/Moderate differentiated	1652	774	878		
Poor differentiation/ Undifferentiated	1837	678	1159		
Unknown	452	233	219		
**T**				4.892	0.027*
T1	2666	1172	1494		
T2	1275	513	762		
**N**				18.399	<0.001*
N0	3550	1478	2072		
N1	391	207	184		
**Stage**				0.251	0.616
Ia	2275	965	1310		
Ib	1666	720	946		
**Surgery**				138.01	<0.001*
Yes	2773	1019	1754		
No/Known	1168	666	502		

*P < 0.05 was considered of significance.

**Table 2:** Uni- and multi-variable analyses for early cardia and non-cardia patients

Characteristics	Univariable analysis		Multivariable analysis		
	Log-rank test	*P*	HR	95%CI	*P*
**Gender**	4.888	0.027*			
Male			Reference		
Female			0.876	0.782-0.981	0.022*
**Age**	87.825	<0.001*			
<65			Reference		
≥65			1.509	1.350-1.687	<0.001*
**Marital status**	47.357	<0.001*			
Married			Reference		
Unmarried/Unknown			1.223	1.101-1.359	<0.001*
**Race**	43.331	<0.001*			
White			Reference		
Others			0.875	0.773-0.990	0.034*
**Tumor location**	116.361	<0.001*			
cardia			Reference		
non-cardia			0.806	0.719-0.904	<0.001*
**Histology**	8.947	0.003*			
Adenocarcinoma			Reference		
Signet ring cell carcinoma			1.328	1.157-1.524	<0.001*
**Grade**	39.294	<0.001*			
Well/Moderate differentiated			Reference		
Poor differentiation/ Undifferentiated			1.284	1.146-1.439	<0.001*
Unknown			0.951	0.804-1.124	0.553
**Stage**	19.028	<0.001*			
Ia			Reference		
Ib			1.536	1.386-1.701	<0.001*
**Surgery**	1400.733	<0.001*			
Yes			Reference		
No/Known			5.523	4.937-6.178	<0.001*

*P < 0.05 was considered of significance.

**Table 3:** Clinical characters for patients with early cardia cancer and non-cardia cancer after PSM

	N	cardia	non-cardia	chi-square	*P*
**Gender**				0.109	0.742
Male	1491	749	742		
Female	647	320	327		
**Age**				1.360	0.244
<65	669	322	347		
≥65	1469	747	722		
**Marital status**				1.123	0.289
Married	1284	630	654		
Unmarried/Unknown	854	439	415		
**Race**				0.003	0.954
White	1783	891	892		
Others	355	178	177		
**Histology**				0.626	0.429
Adenocarcinoma	1876	944	932		
Signet ring cell carcinoma	262	125	137		
**Grade**				2.379	0.304
Well/Moderate differentiated	946	467	479		
Poor differentiation/ Undifferentiated	964	477	487		
Unknown	228	125	103		
**Stage**				1.206	0.272
Ia	1255	640	615		
Ib	883	429	454		
**Surgery**				0.218	0.640
Yes	1474	732	742		
No/Known	664	337	327		

*P < 0.05 was considered of significance.
